# On the Condition of the Nails in Fractured Limbs

**Published:** 1842-01

**Authors:** 


*On the Condition of the Nails in Fractured Limbs.*—Dr. Pitschaft mentions, as a remarkable fact, that the nails do not grow during the union of a fracture, and that the commencement of growth in them is proof that the fracture has united. Dr. Lesler is mentioned as the first who ever observed this. Does this arrest of growth depend merely on the fracture of the limb, or is it, as the editor of the British and Foreign Review suggests, but indirectly connected with the fracture, depending on the well known principle, that the growth of the various horny tissues depends on the amount of waste to which they are exposed. The subject should be investigated.—*New York Medical Gazette.*

